# Complete thoracoscopic resection for TXN2aM0 primary lung cancer: a case series with 5-year follow-up

**DOI:** 10.1186/s44215-025-00228-z

**Published:** 2025-10-14

**Authors:** Daiki Yoshikawa, Takeshi Kawaguchi, Ryo Miyata, Keiji Yamanashi, Maiko Takeda, Akihiko Yoshizawa, Takao Osa, Kazuya Tanimura, Shigeto Hontsu, Shigeo Muro, Sachiko Miura, Mitsuharu Hosono, Masatsugu Hamaji

**Affiliations:** 1 Department of Thoracic and Cardiovascular Surgery, Nara Medical University, 840 Shijo-Cho, Kashihara, Nara 634-8511 Japan; 2 Department of Diagnostic Pathology, Nara Medical University, Nara, Japan; 3 Department of Respiratory Medicine, Nara Medical University, Kashihara, Japan; 4 Department of Radiation Oncology, Nara Medical University, Kashihara, Japan

**Keywords:** Lymph node metastasis, Primary lung cancer, Surgery, Mediastinal lymphadenopathy, Thoracoscopic resection

## Abstract

Management of resectable TXN2aM0 primary lung cancer is controversial, and data regarding the long-term outcomes and guideline recommendations for such cases are lacking. We present the characteristics and long-term outcomes of four patients who underwent complete thoracoscopic resection of TXN2aM0 primary lung cancer. All patients experienced an uneventful postoperative course and survived for 5 to 10 years postoperatively; however, one patient experienced local recurrence at 2 years postoperatively and one patient experienced local recurrence at 5 years postoperatively. Our limited experience suggested that long-term survival may be achieved with complete resection of TXN2aM0; however, late recurrence and local recurrence are possible.

## Background

Isolated mediastinal lymphadenopathy with or without fluorodeoxyglucose (FDG) avidity may be occasionally encountered in clinical practice, and histological information is crucial to patient management. According to the National Comprehensive Cancer Network (NCCN) guidelines for carcinoma of unknown primary sites, adenocarcinoma and other epithelial malignancies limited to the mediastinum of patients older than 50 years of age should be treated using the same methods recommended for non-small cell lung cancer (NSCLC) [1, 2]. However, to the best of our knowledge, data regarding the long-term survival of patients who have undergone complete resection of a metastatic mediastinal lymph node with a radiologically occult primary tumor that was pathologically confirmed as NSCLC are lacking [3]. This report describes the characteristics and long-term outcomes of patients who underwent complete resection of isolated mediastinal lymph node metastasis that was pathologically diagnosed as radiologically occult NSCLC. Pathological staging was based on the Union for International Cancer Control TNM Classification of Malignant Tumors (9th Edition).

## Case presentation

### Patient A

**Fig. 1:**
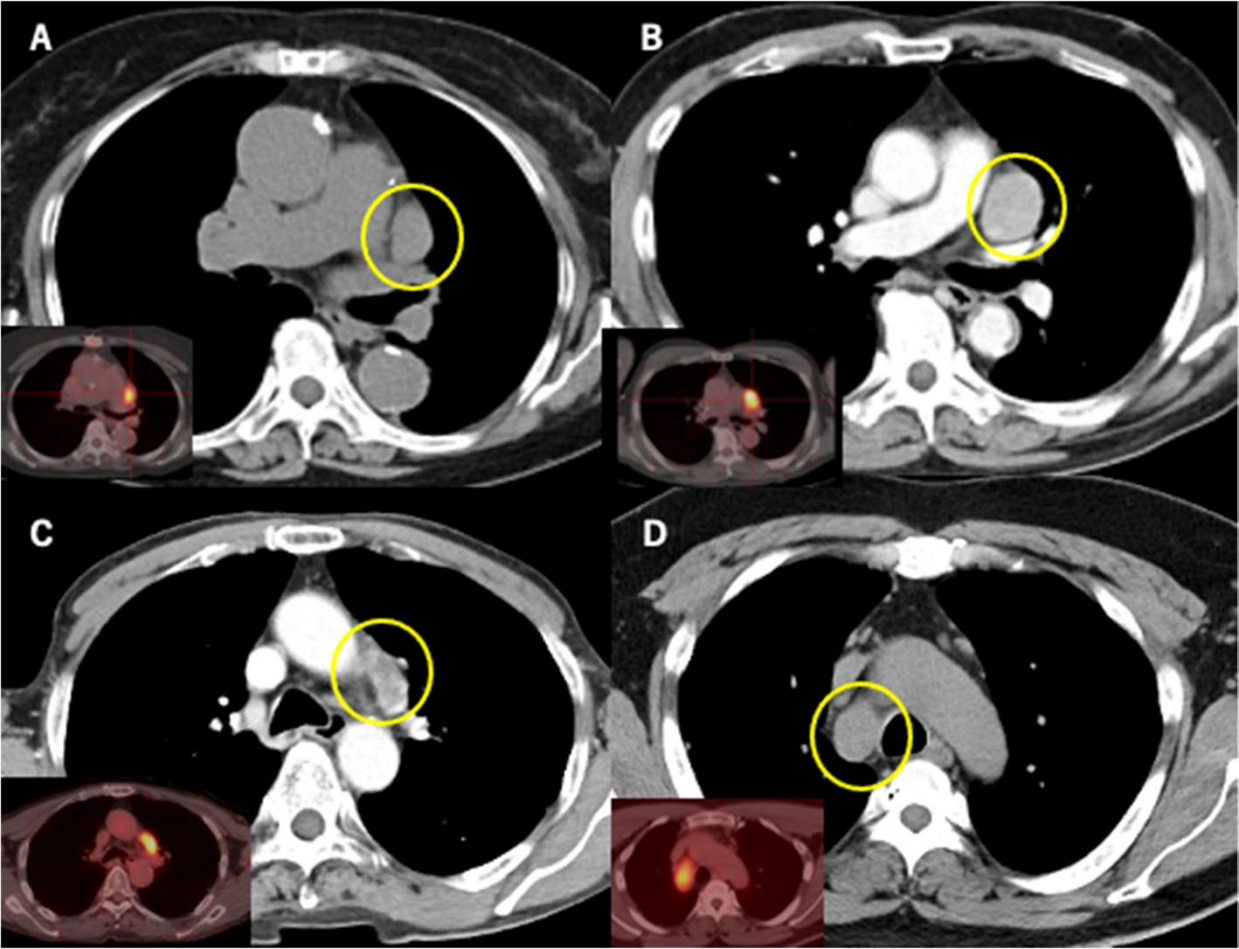
Preoperative CT (yellow circles) and PET images of Patient A (**A**), Patient B (**B**), Patient C (**C**), and Patient D (**D**)

**Fig. 2:**
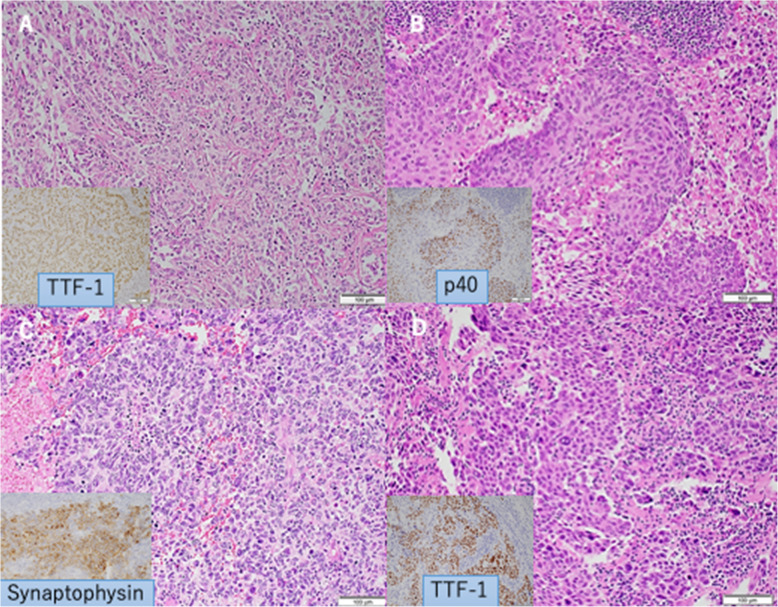
Histopathology and immunohistochemistry from Patient A (**A**), Patient B (**B**), Patient C (**C**), and Patient D (**D**)

A 61-year-old man with hypertension and a 20-pack-year smoking history underwent induction chemoradiotherapy, complete resection, and postoperative adjuvant chemotherapy for rectal adenocarcinoma (ypT3N0M0) with no abnormal findings in chest computed tomography (CT). During the first year postoperatively, recurrence was not detected; however, the carcinoembryonic antigen level was elevated. Chest CT showed an enlarged subaortic lymph node, and FDG avidity was observed using positron emission tomography (PET), as in Fig. 1A. Initially, lymph node metastasis of rectal cancer or malignant lymphoma was suspected. Therefore, a thoracoscopic excisional biopsy was performed. Intraoperatively, we identified an enlarged subaortic lymph node and performed complete resection with negative margins. The postoperative course was uneventful. Immunohistochemical study results indicated positivity for TTF-1, CK7, and napsin-A and negativity for p40, CK20, CDX2, synaptophysin, chromogranin-A, and CD56, suggesting metastatic adenocarcinoma (solid-predominant adenocarcinoma) from radiologically occult primary lung cancer (Fig. 2A). The referring physician chose not to administer adjuvant therapy. The patient was alive without evidence of disease at 7 years postoperatively.

### Patient B

An inflammatory nodule in the left upper lobe was suspected in a 67-year-old woman with a 20-pack-year smoking history, hypertension, coronary artery disease, and chronic renal failure requiring hemodialysis. Although CT showed a nodular lesion that regressed over 2 years, a subaortic lymph node was enlarged with FDG avidity (Fig. 1B). A thoracoscopic excisional biopsy was performed, and the enlarged lymph node was completely resected. The patient experienced an uneventful postoperative course. Pathological examination revealed moderately differentiated squamous cell carcinoma; immunohistochemical results were positive for P40 (Fig. 2B). Adjuvant therapy was not administered due to comorbidities. Local recurrence developed in the mediastinum at 5 years, and the patient received 60 Gy of radiotherapy. Radiation pneumonitis was managed with steroids. At 10 years postoperatively, the patient died from brain metastasis.

### Patient C

A 77-year-old man with a 20-pack-year smoking history underwent distal gastrectomy and adjuvant chemotherapy for stomach adenocarcinoma without any neuroendocrine differentiation (pT3N1M0). Nine years later, surveillance CT showed subaortic lymph node enlargement with FDG avidity with no abnormal findings in the lungs (Fig. 1C). Thoracoscopic resection revealed carcinoma with neuroendocrine features. Immunohistochemical study indicated positivity for TTF-1 and synaptophysin (Fig. 2C). Molecular studies were negative for EGFR mutations, ALK rearrangements, and ROS1, with negative PD-L1 expression. Two years later, CA19-9 elevation and FDG uptake in the left hilar node were detected. Local recurrence was treated with 70 Gy of radiotherapy, but progressive disease with left recurrent nerve palsy developed. The patient died from pneumonia resulting from left recurrent nerve palsy 5 years postoperatively.

### Patient D

A 58-year-old man with pneumoconiosis and a 69-pack-year smoking history presented with back pain. Imaging revealed a paratracheal lymph node (station 4R) with FDG avidity (SUV 11.4), as in Fig. 1D. EBUS-guided biopsy showed adenocarcinoma. Thoracoscopic resection confirmed adenocarcinoma; immunohistochemical study showed strong positivity for TTF-1 (Fig. 2D). Molecular testing was negative for EGFR, ALK, and ROS1; PD-L1 expression was high (50%). The patient received adjuvant radiotherapy (66 Gy), six cycles of carboplatin/paclitaxel, and 16 cycles of durvalumab. He remained disease-free at 5 years.

## Discussion

This case series highlights important but limited findings. Data on patients undergoing complete resection of TXN2aM0 primary lung cancer are rare in databases. Major findings include the potential for long-term survival after thoracoscopic resection of mediastinal metastasis from occult lung cancer and the variability in adjuvant therapy use. Among four patients, two remained recurrence-free long term, while two developed local recurrence. Notably, Patient A survived 7 years without adjuvant therapy, Patient B had local recurrence treated with radiotherapy but ultimately died from brain metastasis, and Patient C died from pneumonia secondary to left recurrent nerve palsy due to local recurrence, whereas Patient D remained disease-free following combined therapy.

Histological confirmation was not always feasible during clinical courses of our patients. Specifically, the nodule in the left upper lobe of Patient B was not confirmed histologically, therefore the possibility of spontaneous regression was not completely excluded. In Patient C, FDG-avid left hilar node with elevated CA 19–9 may not be a local recurrence of TXN2aM0 lung cancer but a recurrence of stomach adenocarcinoma, although it was unlikely.

Specific guidelines for adjuvant therapy post-resection of TXN2aM0 NSCLC are lacking. Current NCCN guidelines suggest definitive chemoradiotherapy plus durvalumab or Osimertinib for unresectable T1-3N2M0 and adjuvant therapy for resectable T1-2N2M0 [1, 2]. Our cases reflect diverse real-world management.

Half of the patients experienced local recurrence, but the survival following recurrence was relatively prolonged, suggesting possible indolent tumor behavior. A complete 5-year follow-up is warranted in such cases, although no previous study reported complete follow-up of 5 years [4].

Because of the rarity of resected TXN2aM0 cases, statistical comparison with larger surgical cohorts (T1–4N2aM0 NSCLC) was not feasible. However, a previous study of patients who underwent radiotherapy for stage III NSCLC with either occult or known primary lesions suggested that TXN2M0 patients may have improved 5-year overall survival (61.6%) compared with T1–4N2M0 patients [3]. This limitation underscores the need for future multi-institutional studies.

In conclusion, complete thoracoscopic resection for TXN2aM0 primary lung cancer may be associated with long-term survival. Long-term follow-up is essential, and adjuvant therapy may be considered if tolerated. Further studies are needed to clarify its benefit.

## Data Availability

Not available.
